# Indian Association of Pediatric Surgeons-Presidential Address delivered at 36^th^ annual conference, New Delhi, October 2010

**DOI:** 10.4103/0971-9261.72432

**Published:** 2010

**Authors:** Ashoke K. Basu

**Affiliations:** President, IAPS Kolkata, India. E-mail: ashokekbasu@rediffmail.com

Honourable State Minister of Health and Family Welfare, Government of India, Mr. Dinesh Trivedi, Prof. D. K. Gupta, Organizing Chairman and President of FAPSS, Dr. Rasik Shah, Honorary Secretary, IAPS, Dr. Ashley D’Cruz, President Elect IAPS, past presidents, chairpersons and secretaries of all the sections and chapters of the association, invited guests, senior members from neighboring countries, delegates, ladies and gentlemen,

I feel honoured, happy and privileged to stand before this August gathering on this auspicious occasion of inauguration of the 36^th^ Annual Conference of the IAPS being held jointly with the World Congress of Federation of Association of Pediatrics Surgery. I take this opportunity to thank all my teachers, namely, Prof. P. Upadhyaya, Prof. D. K. Mitra, Prof. Subir K. Chatterjee, Prof. D. Basak, Prof. James Lister, for their generosity, kindness and perseverance. It is by virtue of their assiduousness that I stand here on this platform today. I also express my regards and thanks to my late parents who are still my idol. I am also thankful to my friends and family, especially my wife, for their unstinted support. She always knew a better way to do everything standing at the head end of the operating table.

Pediatric surgery in India started its journey at the pediatric ward of the Medical College in Calcutta of which Dr U. C. Chakrabarty was made the Surgeon-in-Charge in 1946. In 1965 at Mumbai, 12 enthusiastic surgeons interested in surgery for children formed the Chapter of Association of Surgeons of India. The first mid-term conference was held in Mumbai and the second one was held in Kolkata. Such meetings have progressed to the present 36^th^ Annual Conference. The hard work and determination of the founding fathers has paved the way for the growth of pediatric surgery in India. In this journey, we have overcome many hurdles. After establishment of safe surgery for older children, the next challenge was neonatal surgery. Tertiary care centers for newborn have now become available in all the states of the country. Some of the centers can be compared with the best in the world. The progress of endoscopic surgery for children has been phenomenal and now we are seriously embarking on safe neonatal endoscopic surgery. However, our services are yet to reach the rural population of the country. This is where the association comes in, and we, the members, have a major role to play in improving this situation.

The growth of pediatric surgery in India has been hampered by lack of finance, deficiencies in teaching and training, and most importantly, improper deployment of trained surgeons.

The central and the state governments have succeeded in providing finance and infrastructure to a large extent but these are still inadequate. Other than government aid, we need to actively participate in establishing the system of insurance for pediatric surgical patients. The schemes like National Raashtriya Swastha Bima Yojana for below poverty line families started jointly by central and state governments and the outsourcing of medical services to public–private joint ventures for government employees of some states are steps in the right direction.

It is extremely unfortunate that the products that cover congenital defects are not popularized by the insurance companies of India. We are in communication with the IRDA so that the insurance companies extend the benefits of medical insurance at the least to pediatric surgical patients for diseases which may have congenital predisposition but are not obvious at birth. Members of our executive committee are also in touch with competent government authorities for formulation of suitable insurance schemes for pediatric population. It is heartening to see some of the states like Karnataka and Tamil Nadu formulating specific policies to cater to pediatric patients. I request the office bearers and the members of all the chapters to take up the issue with the local authorities and try to extend the financial benefits to the deserving patients. Better coverage of the population will ensure development of superior equipped centers in the peripheral areas.

Better teaching and training of a pediatric surgeon and his proper deployment are related problems. Pediatric surgery is taught to medical students at various levels by general surgeons in many of the medical colleges. This creates a dual problem of improper teaching to students who either lose interest or do not develop any interest at all in pediatric surgery, and hindrance in creation of pediatric surgical positions in the teaching institutions. The association has been pursuing the Medical Council of India for rectification of this matter since 2002. I have personally taken up the matter with the erstwhile council and have also communicated with members of its current board. It is reassuring to learn that the council is in the process of conducting a survey in the various states for reorganization of the medical curriculum. We have provided the authorities with copies of all past communications and have made a fresh appeal. I will again impress on the members to persuade the local authorities for creation of the post of pediatric surgeons in all district hospitals, state general hospitals and sub-divisional hospitals for the benefit of children and the trained professionals. I would like to draw attention of the young and freshly qualified pediatric surgeons to the field survey conducted by PGI, Chandigarh, and CMC, Vellore, which has shown that the surgeons working at peripheral areas, both in mission hospitals and in private, have more number of patients and are generally more satisfied in their work life. While the new generation of pediatric surgeons may have some initial difficulties, I am confident that they will overcome these quickly and efficiently. I appeal to these youngsters to bear in mind that India needs 2200 pediatric surgeons to properly treat the entire pediatric population and there are only a thousand of us. Hence, there is a tremendous growth potential in this speciality. I will also encourage the senior surgeons to help their juniors to take up different speciality based pediatric surgeries like thoracic surgeries, neurosurgeries, urology, etc. We also need to consider conferring clinical honorary designation to doctors working in the peripheries in an attempt to keep them involved with the teaching. Similar systems are in vogue in the West.

Dear members, the activities of our esteemed association have progressed smoothly and will be evident from the secretary’s report. I am a little concerned about the health of our journal. I would request the board of editors along with the Editor-in-Chief to put in some extra effort to publish the issues on time.

As a president, I had the honor to open one new chapter of our association and the opportunity to interact with a large number of pediatric surgeons from our various chapters. I was always impressed by their ingenuity and dexterity to work in adverse situations. I congratulate all of you for your commendable work. There is nothing better than helping a child in need or, when one cannot, of intensely studying to make better those whom we have not learnt to cure.

We are privileged to serve the noble profession of pediatric surgery, and I feel proud to have served as your president. I thank all the members for their full hearted cooperation. I thank the members of the executive committee for their excellent support and timely suggestions. I sincerely thank the Hony secretary of the association who has sent me thousands of mails and uncountable calls; together, we have made the telecom industry richer. Thank you, Rasik, working with you has been a pleasure. My special thanks to Prof. D. K. Gupta who has not only made this world congress possible, but has also not left any stone unturned to make it a grand success. I would also like to thank the delegates without whose presence and active participation, no conference can be a success. I wish the association the very best. Long live IAPS. Jai Hind!

